# Effect of Raw Chickpea in the Broiler Chicken Diet on Intestinal Histomorphology and Intestinal Microbial Populations

**DOI:** 10.3390/ani12141767

**Published:** 2022-07-10

**Authors:** Anna Danek-Majewska, Małgorzata Kwiecień, Wioletta Samolińska, Danuta Kowalczyk-Pecka, Bożena Nowakowicz-Dębek, Anna Winiarska-Mieczan

**Affiliations:** 1 Institute of Animal Nutrition and Bromatology, University of Life Sciences, Akademicka Street 13, 20-950 Lublin, Poland; anna.majewska@up.lublin.pl (A.D.-M.); wioletta.samolinska@up.lublin.pl (W.S.); anna.mieczan@up.lublin.pl (A.W.-M.); 2 Department of Zoology and Animal Ecology, University of Life Sciences, Akademicka Street 13, 20-950 Lublin, Poland; danuta.pecka@up.lublin.pl; 3 Department of Animal Hygiene and Environmental Hazards, University of Life Sciences, Akademicka Street 13, 20-950 Lublin, Poland; bozena.nowakowicz@up.lublin.pl

**Keywords:** chickpea, broiler chickens, intestinal histomorphology, intestinal microbial populations

## Abstract

**Simple Summary:**

Raw chickpea seed (CPR) can be an alternative source of cheaper native proteins in the diet of chickens, replacing the standard soybean meal. The present results were obtained in a study on the addition of raw chickpea seeds to replace 50% of soybean meal (SBM) protein in an experimental feed mixture for broiler chickens. We investigated the effect of the substitution on the intestinal histomorphology, intestinal microbial population, and health of broiler chickens.

**Abstract:**

The aim of the study was to determine the effect of partial replacement of SBM protein with CPR-derived protein in the broiler rearing period from 22 to 42 days of age on the intestinal histomorphology and the composition of the intestinal microbial population. Male broiler chicks aged 1 day were assigned to two groups with different nutrition schemes (*n* = 100 in each, 5 cages of 20 chicks in each). The chickens were reared for 42 days. All birds were fed isonitrogenous and isoenergetic diets: starter (1 to 21 d), grower (22 to 35 d), and finisher (36 to 42 d). From rearing day 22, different diets were provided to the birds: the SBM group received feed with 100% soybean meal protein, and the diet of the CPR group the protein originated from soybean meal was replaced by 50% chickpea protein. The study results indicated a significant impact of the inclusion of CPR in the diet on the basic intestinal structures (thickness of myenteron: submucosa, jejunum and duodenum mucosa, and jejunum transversal lamina). The addition of CPR led to shortening of intestinal villi, an increase in villus thickness, and reduced intestine absorptive surface in the duodenum and jejunum. The CPR group exhibited a significantly lower villus length-to-crypt depth ratio in the jejunum (*p* < 0.001). The inclusion of chickpeas in the diet increased the total count of mesophilic bacteria and coliforms in the intestinal contents (*p* < 0.05). In summary, it has been demonstrated that the inclusion of CPR in the diet induced considerable disturbances in metabolism and intestinal structure. Although CPR is a cheap protein source, its use in poultry diet does not ensure development of the intestinal structure comparable to that in the case of an SBM-only diet.

## 1. Introduction

Human and animal diseases caused by *Enterobacteriaceae* are a global epidemiological problem [1,2]. *Enterobacteriaceae* may be, among others, carried by poultry; this may lead to infections, and the consequence may be contamination of meat products by these bacteria [3]. One of the key elements determining the profitability of poultry production is the maintenance of proper intestinal integrity. The gastrointestinal microbiota comprises several hundred bacterial species, mainly anaerobic ones [4], which may exert a positive or pathogenic effect on the organism. The species composition and bacterial counts vary depending on the animal species and age, the fragment of the gastrointestinal tract and its pH, or nutritional and environmental factors [5]. *Firmicutes*, *Bacteroidetes*, and *Proteobacteria* account for over 90% of bacteria present in the chicken microbiome. The most abundant bacteria identified in the poultry gastrointestinal tract represent the genera *Clostridium*, *Ruminococcus*, *Lactobacillus*, and *Bacteroides* [6]. Particularly noteworthy are Lactobacillus or Bifidobacterium bacteria, which not only stimulate the gastrointestinal tract functions but also inhibit the growth of pathogenic microorganisms, e.g., *Escherichia coli* or *Clostridium* [7,8]; they are also reservoirs of antibiotic resistance genes [9]. Special epidemiological importance is ascribed to multiresistant strains, hence the necessity of identification of factors that trigger the mechanism of bacterial resistance to chemotherapeutic agents [10].

Nutrition plays an important role in the determination of the profitability of animal production, and protein is one of the main ingredients influencing the costs of feed mixes for poultry [11]. As shown in previous research, raw chickpea seeds (CPR) seem to be an alternative source of native protein which is cheaper than soybean meal (SBM) [12,13,14]. However, a negative characteristic of CPR seeds is the presence of antinutritional factors (ANF), which may limit the absorption and digestibility of nutrients and impair the absorption of certain components, e.g., Ca, P, or Fe [15]. The negative effects of ANF may also lead to hepatotoxicity, toxic nephrosis, and intestinal mucosa damage [16]. By binding to the receptors of intestinal mucosa epithelial cells, ANF damage mucosa cells and disrupt digestive processes [17]. They can also alter the immune function of intestines, reduce the generation of endocrine cells and production of intestinal hormones, and inhibit the growth of intestinal lumen bacteria [18].

Reactive oxygen species (ROS) may be produced in the organism as a result of metabolic changes or the action of certain environmental factors. An imbalance between ROS production and the organism’s defense system, i.e., the biological detoxification system, leads to cell damage. Hence, natural substances preventing adverse oxidative reactions in the organism and serving as a good source of protein are being investigated [19]. Some plants contain antioxidant compounds (tocopherol, flavonoids, or carotenoids) and are concurrently rich in proteins, metal-chelating amino acids, and hydrogen donors [20]. Chickpea is a source of cheap protein, vitamins, and minerals that can be used as a supplement in animal nutrition. As in the case of other legumes, chickpeas are perceived as a source of compounds with antioxidant properties [14,21,22]. Chickpea seeds are rich in such valuable amino acids as arginine (involved in detoxification), leucine, and lysine. High lysine content is an important element in a balanced diet and is probably more important than total protein, as it is an important dietary legume supplement of cereals, which are known to be lysine deficient [23]. Arginine is considered an important part of the diet, as it has a positive effect on weight gains [24]. It is also a precursor to the synthesis of many nitrogen-containing compounds, e.g., nitric oxide. This role of chickpea-derived arginine has been the subject of research in particular in recent years, as it is an important signal in many physiological and pathological processes in animals [22].

Given the major adverse effects of antinutritional substances on utilization of minerals and gastrointestinal function, a diet based on raw chickpea seeds (CPR) is expected to exert an adverse effect on intestinal development. Many studies [14,21,22] have reported a beneficial effect of CPR on growth parameters and slaughter yield in poultry, but there are no studies on its effect on intestinal histomorphology and the population of intestinal microorganisms. No experiments have focused on the development of the gastrointestinal tract induced by the dietary use of CPR. Therefore, we have proposed a hypothesis that the basic source of dietary protein may influence the histomorphometric parameters of intestines and the composition of intestinal microbiota in chickens. The aim of the study was to investigate the effect of partial replacement of SBM protein with CPR-derived protein in the rearing period from 22 to 42 days of age on the intestinal histomorphology and microbiota composition.

## 2. Materials and Methods

### 2.1. Birds, Diets, and Experimental Design

All procedures used during the research were approved by the Local Ethics Committee for Animal Testing at the University of Life Sciences in Lublin, Poland (Resolution No. 33/2015 of 26 May 2015).

Two hundred one-day-old male Ross 308 chicks were randomly assigned to two groups (*n =* 100 in each; 5 cages of 20 chicks in each) and reared until day 42 with unlimited access to feed and water. The chicks were reared in 1-m^2^ cages in lighting, humidity, and temperature conditions meeting the requirements for Ross 308 chickens [25].

Three types of diets were used: starter (days 0 to 21—crumble), grower (days 21 to 35—pellet), and finisher (days 36 to 42—pellet). In the starter period, the chicks in the control and experimental groups were fed the same diets based on cereal meal, i.e., wheat, corn, and soybean (Table 1) [14]. From day 22, the diets were differentiated with the source of protein as the experimental factor. SBM constituted a 100% protein source in the control group mix (grower and finisher), whereas the protein originated from soybean meal was replaced by 50% chickpea protein in the diet for the experimental group (grower and finisher). The chemical composition of the mixtures is shown in Table 1 [14]. The content of antinutritional factors (tannins and trypsin inhibitors) in the feed mixtures is presented in Table 1 (based on data reported in [14]). All the feed mixtures were mutually isonitrogenous and isoenergetic and balanced in line with the dietary recommendations for Ross 308 chickens [26].

### 2.2. Intestinal Tract Histomorphometrical Analysis

The male broilers were not fed 10 h before slaughter but had constant access to water. Before slaughter, on the morning of rearing day 42, the male broilers were weighed, and 10 birds with body weight representative for each group were selected. After weighing, the male broilers were stunned electrically and slaughtered by decapitation [14]. Two intestine segments (10 mm long each) from the duodenum (10 mm distal to the pylorus) and jejunum (50% of the total length) were sampled from each male broiler chicken. The segments were opened longitudinally along the mesenteric border and pinned flat without stretching in physiological saline on histological cassettes [27]. The samples were fixed in 4% buffered formaldehyde (pH 7.0) for 24 h. Next, they were dehydrated in graded ethanol solutions, fixed, and embedded in paraffin using an STP 120 spin tissue processor (ThermoFisher Scientific, Waltham, MA, USA). Next, 4 μm thick tissue sections were cut with a microtome (Microm HM 360, Microm, Walldorf, Germany) and subjected to histological examination following a procedure described previously [28]. The small intestine wall layers were differentiated efficiently using Masson’s trichrome staining [29].

Microscopic (two-dimensional) images were taken with the use of an AXIOVERT 200M microscope (Carl Zeiss, Jena, Germany) and an AxioCam HRc camera (Carl Zeiss, Jena, Germany). Magnifications of 4×, 10×, 20×, and 40× were used to obtain images of the intestinal structures. The small intestine wall structure was examined using a microscope and graphic analysis software (Olympus cellSens Version 1.5; Olympus, Tokyo, Japan) [30]. The following morphometric parameters in the intestine were evaluated: the thickness of mucosa, submucosa, and myenteron (longitudinal and transversal lamina); the thickness of the villus epithelium, crypt depth (defined as the depth of the invagination between adjacent villi from the bottom of the crypt to the base of villi); crypt width (measured in the middle of the crypt depth); the number of crypts (active: showing mitotic activity and Paneth cells with an open internal space and access to the intestinal lumen; inactive: showing no mitotic activity and Paneth cells with a closed internal space; total: inactive plus active crypts); villus length (from the tip of the villi to the villus–crypt junction); villus thickness (measured in the middle of villus height); the number of villi; and the small intestine absorptive surface [30]. Only vertically oriented villi and crypts were measured.

Meissner and Auerbach plexuses were localized with the use of immunohistochemical reaction with a mouse monoclonal antibody to the 200 kD neurofilament heavy subunit neuronal marker (Sigma-Aldrich, St. Louis, MO, USA, dilution 1:40). After deparaffinization and rehydration, antigen retrieval was achieved by boiling the duodenum and jejunum sections in citrate buffer (pH 6.0) three times for 5 min in a microwave oven (700 W). To block endogenous peroxidase activity, a 3% solution of hydrogen peroxide in methanol (1:1) was applied for 30 min. The sections were blocked in normal serum and incubated with the primary antibody at 4 °C overnight, followed by 30 min incubation with the secondary antibody—biotinylated rabbit polyclonal—and mouse immunoglobulin (Sigma-Aldrich, St. Louis, MO, USA, dilution 1:200) with streptavidin/HRP (DacoCytomation, Glostrup, Denmark, dilution 1:300) and development in 3‘3‘-diaminobenzidine tetrahydrochloride (DAB; DacoCytomation, Glostrup, Denmark) for 15 min at room temperature. Sections that served as a negative control were prepared for immunohistochemical staining with omission of the primary antibody. All the sections were counterstained with the use of Mayer’s hematoxylin.

Microscopic images of the immunohistochemistry reactions described above were further analyzed. The following variables were analyzed for detection of neurofilaments: the cross-sectional area of the nerve ganglion, sphericity, perimeter, mean Feret diameter, and minimal and mean diameters of the ganglion. The Feret diameter was defined as the distance between two parallel planes restricting the object perpendicular to that direction [31].

### 2.3. Microbiological Analysis of Intestinal Contents

Pooled samples of intestinal contents (from the jejunum and iliac intestines) were collected from both groups for microbiological assays. From each of the groups, 20 g portions were taken and placed in sterile bottles containing the dilution fluid. The samples were homogenized for 5 min and sedimented for another 15 min. The suspension was diluted, and 0.1 mL of the solution was transferred onto microbiological media. The following parameters were determined in the experimental material: the total count of aerobic mesophilic bacteria and the total count of bacteria on enriched agar medium (BTL Ltd., Łódź, Poland), the total count of fungi and molds on Sabouraud agar (BTL Ltd., Łódź, Poland), and the total count of coliform bacteria on Endo-Les medium (BTL Ltd., Łódź, Poland). The colonies were counted with the use of a Scan 300 automatic counter (Interscience, Saint Nom la Brétèche, France). The results, expressed as colony-forming units (log cfu/g), are presented in the table.

### 2.4. Identification of Enterobacteriaceae Species from the Cecum and Cloaca

The material for the microbiological analyses was collected from the cloaca and cecum once with the use of sterile swabs. LB nutrient broth (BioMaxima, Lublin, Poland) incubated at 37 °C for 24 h was used to multiply the bacteria in the laboratory conditions. Next, the material was transferred to SalmonellaShigella (S-S) agar (BioMaxima, Lublin, Poland) and grown at 37 °C for 24 h. After incubation on a selective medium, bacterial colonies originating from the chickens were selected for further identification and analysis. The entire collection of the isolated strains was analyzed biochemically. Each strain was identified using the commercial API 20E test kit (bioMérieux, Warsaw, Poland).

### 2.5. Analysis of Sensitivity to Chemotherapeutics

The bacterial sensitivity to chemotherapeutic agents was determined with the disk diffusion method [32] on Mueller–Hinton agar (BioMaxima, Lublin, Poland), using Oxoid disks (Hampshire, England). The method facilitated rapid determination of the efficacy of the chemotherapeutic agent by measurement of the diameter of the inhibition zone resulting from the diffusion of the agent into the disk surrounding area. The following chemotherapeutic agents were used to determine the drug susceptibility of the isolates: chloramphenicol (C 30 μg/mL), tetracycline (TE 30 IU), trimethoprim/sulfamethoxazole (TMP/SXT 1.25 + 23.75 µg/mL), streptomycin (S 10 IU), nitrofurantoin (FM 300 IU), and ampicillin (AM at a concentration of 10 µg/mL). The plates were incubated at 37 °C for 24 h; next, they were examined, and the diameters of complete inhibition zones were measured. The results were interpreted in accordance with the manufacturer’s recommendations. The diameters of the inhibition zones were measured, and the isolates were classified as sensitive (S), moderately sensitive (MS), and resistant (R).

### 2.6. Statistical Analysis

Statistical analysis was performed using Statistica software, version 13.3.721.0 (StatSoft Poland Sp. z o.o., Kraków, Poland, 2022, www.statsoft.pl) (accessed on 18 May 2022). The normality of data and homogeneity of variances were tested using the Shapiro–Wilk and Brown–Forsythe tests, respectively. The data obtained were analyzed statistically using Student’s *t*-test and the nonparametric Mann–Whitney U test. Variability in the data was expressed as the standard error of the mean (SEM), and *p* < 0.05 was considered to be statistically significant. The antimicrobial susceptibility data are expressed as percentages (category: sensitive, moderately sensitive, and resistant), and the differences between the treatments were compared using the nonparametric χ^2^ test. *p* < 0.05 was considered statistically significant.

## 3. Results

### 3.1. Gastrointestinal Tract Morphology

The addition of CPR exerted a negative effect on the histomorphometric parameters in the duodenum and jejunum (Table 2 and Table 3). The transversal lamina thickness in the duodenum was reduced (by 43%) in the CPR group compared to the SBM variant (Table 2). The CPR supplementation in the diets reduced the submucosa thickness by 12.7% and the mucosa thickness by approx. 14% relative to SBM. Moreover, there was a significant increase in the height and total number of enterocytes (by 40.6% and 39.6%, respectively) in the CPR group. The results revealed that the intestinal villi in the duodenum were shortened (by 7%), while the thickness of the villus epithelium increased by 24% in the CPR group. The intestine’s absorptive surface decreased (*p* < 0.001), and the number of closed crypts increased (*p* = 0.025) (Table 2).

The addition of CPR in the diets of the broiler chickens reduced the thickness of the longitudinal and transversal lamina, submucosa, and mucosa in the jejunum by 60.5%, 52%, 43%, and 20%, respectively, compared to the SBM group (Table 3). In the jejunum of the CPR-supplemented group, the villi were significantly shortened, but the villus thickness increased. Additionally, a significantly lower villus length-to-crypt depth ratio and a reduced intestinal absorptive surface were observed in the CPR group (Table 3).

There was no effect of CPR on the histomorphometric parameters of the nerve plexuses in the duodenum of the broiler chickens at the age of 42 days (Table 4). The addition of CPR caused a decrease in almost all histomorphometric parameters of the jejunum nerve plexuses except for the sphericity in the Auerbach plexus (Table 5). An increase in perimeter (*p* = 0.003) and mean Feret diameter (*p* = 0.003) and a decrease in sphericity (*p* < 0.001) were recorded in the jejunum *Meissner plexus* (Table 5).

### 3.2. Concentration of Microorganisms in Intestinal Contents

The results of the microbiological analysis of intestinal contents (from the jejunum and iliac intestines) are presented in Table 6. They indicate that the use of CPR as a substitute for SBM in the diet increased the total count of mesophilic bacteria and coliforms in the intestinal contents in comparison to the SBM group (*p* < 0.05). In turn, the inclusion of CPR in the diet did not affect the total count of intestinal bacteria and fungi (*p* > 0.05).

### 3.3. Enterobacteriaceae Species in the Cecum and Cloaca of Broiler Chickens

The isolates obtained from the broiler chicken belonged to *Enterobacteriaceae* and were represented by eight species in the cloaca and cecum (Table 7). Eight species of *Enterobacteriaceae* were identified in the material from the SBM chickens, and two taxa were identified in the CPR group, i.e., *Citrobacter braakii* and *Proteus mirabilis*, which were abundant in the cecum and cloaca (Table 7).

### 3.4. Sensitivity of Enterobacteriaceae Strains to Chemotherapeutics

*Enterobacteriaceae* strains resistant to chemotherapeutics were found in both study groups (Table 8). Since nearly all *Enterobacteriaceae* may have epidemiological significance in certain conditions, their potential transfer to the environment via the gastrointestinal tract is of sanitary importance.

## 4. Discussion

Animal health is largely determined by the proper function of the gastrointestinal tract, mainly by the morphological integrity of intestines and appropriate abundance and structure of intestinal microbiota [33]. The development of the intestinal microstructure and nutrient transport are influenced by, e.g., the composition of the feed mixture and the presence of nutrients and antinutritional factors in the gastrointestinal tract. Protein is a key regulator of growth, reproductive performance, and gastrointestinal tract development in poultry [34,35]; therefore, protein utilization efficiency will partly depend on the characteristics of the gastrointestinal tract [36]. Various studies have shown that low-protein diets cause slower histological regeneration of intestines [37]. A long-term low-protein diet does not induce changes in intestinal villi, whereas chronic feeding may lead to histological alterations. In turn, longer villi, a larger cell surface area, and a high number of cells undergoing mitosis have been observed in protein deficiency conditions [38]. Hypotrophic histological changes in the intestine may indicate nutritional imbalance of the diet. It has been reported that CPR contains very small amounts of sulfur amino acids (methionine and cysteine) but is rich in lysine [17]; therefore, animal diets should be isonitrogenous and isoenergetic. This type of diet was administered to the chickens in the present experiment. However, the results of our study diverge from findings reported by other researchers, as the inclusion of CPR in the diet of the broiler chickens exerted a significant negative effect on the intestinal histomorphometry.

The study investigated the effect of the partial substitution of SBM protein with CPR protein on the intestinal histomorphology and the population of intestinal microorganisms in broiler chickens. To the best of our knowledge, most reports on CPR supplementation in poultry describe its effect on production performance and slaughter yield parameters [14,21,22], but there are no studies on the effect of CPR on the development of the gastrointestinal tract, which determines the development of the organism in birds.

The morphology of the small intestine is the main indicator of normal intestinal histology and, therefore, the health of chickens. The absorption of nutrients in this part of the gastrointestinal tract is facilitated by its large surface [39]. The functional state of the intestine is mainly determined by the villus height and crypt depth, which play an important role in the final stage of digestion and nutrient assimilation [40], and by the villus length-to-crypt depth ratio [41].

The present experiment showed that the CPR supplementation in the chicken diet resulted in the shortening of the villi in both sections with no effect on the basic parameters of the crypts. This may suggest that the rate of cell proliferation taking place in the crypt and determination of the renewal of the intestinal epithelium were unchanged regardless of the type of nutrition. In turn, the shortening of the villus length may be a result of excessive cell shedding at the villus apex as part of the renewal of the intestinal epithelium, which takes place every few days. Apical cell shedding is probably caused by rapid autophagy and apoptosis of enterocytes exposed to direct contact with the toxic and antinutritional factors contained in CPR. It should be emphasized that the mucosa is most exposed to the effects of toxins and pathogens [42]. In this experiment, no specific proliferation- and apoptosis-related analyses were carried out, but the present results indicate the need for further investigation of this issue.

The shortening of villi evidently leads to reduction of the intestine absorptive surface [43]. Probably the increased width of villi and definitely the increase in the height and number of enterocytes compensate for this effect. Changes in the morphology of enterocytes may result in impairment of nutrient transport by these cells responsible for absorption. The impairment of the absorption process leads to poorer nutritional status of the entire organism. It is also unknown whether changes in morphology affect the production of brush border enzymes. Enterocytes are also involved in immune defense functions. This aspect should also be further investigated.

The histological observations carried out in the present study showed that the inclusion of raw CPR in the diet had an impact on the gastrointestinal tract, i.e., it mainly reduced the absorptive surface in the duodenum and jejunum, possibly in response to the antinutritional factors present in the seeds. The negative effect (significant reduction) of the inclusion of CPR in the chicken diets on the basic intestinal structures (thickness of myenteron: submucosa, jejunum and duodenum mucosa, and jejunum transversal lamina) may lead to abnormal intestinal motility. Additionally, the inclusion of CPR in the diet may affect the intestinal flora and immune system of the host. This may be related to the altered activity and number of crypts where Paneth cells secrete antimicrobial peptides [44]. Moreover, the CPR supplementation exerted an effect on the nerve plexuses, depending on their type and location in the gastrointestinal tract. Overall, in the CPR group, the Auerbach plexus in the jejunum was reduced by half and the *Meissner plexus* was approx. 1.3-fold larger than in the SBM group. As this issue was not investigated in the present study, it can only be assumed that the changes in ganglia responsible for myenteron contractions and the reduction of the lamina thickness may lead to disturbances in intestinal content transport and mixing. Disturbance in passages as a result of myenteron thinning may cause prolonged retention of chyme in the intestinal lumen, while weak mixing contractions may result in impaired digestion and decomposition of food and disturb absorption. Retention and insufficient chemical processing of food may have an effect on the composition and functions of intestinal bacterial flora.

Animal feed and rearing environments determine the microbiota of the gastrointestinal tract. Appropriate quantitative and qualitative structure of the intestinal microbiota (known as eubiosis) has a considerable impact not only on nutrient digestion and absorption but also on the metabolism and immune functions of the entire organism. This multidirectional impact on the organism is reflected in the nutritional status, health, and production performance of livestock animals. Since some species or genera of intestinal bacteria may have pathogenic properties, it is essential to ensure quantitative dominance of bacteria that exert a favorable effect on intestinal processes and a health-enhancing effect on the animal organism. The different nutritional preferences of microorganisms or the animal’s preferences for consumption of specific ingredients have an effect on the diversity of intestinal microbiota [45,46,47,48]. In the present study, the inclusion of chickpea seeds in the diets for broiler chickens changed the composition and concentration of the gastrointestinal microbiota.

There are only few literature reports on the potential modifying effect of chickpeas on the gastrointestinal microbiota in broiler chickens. Ciurescu et al. [49] used increasing doses of chickpeas as a substitute for SBM in diets for broiler chickens. However, in contrast to the present results, they did not find an influence of chickpeas on coliform counts in the cecum. The increase in the total counts of mesophilic bacteria and coliforms in the intestinal contents observed in the present study may be associated with the presence of oligosaccharides in chickpea seeds, which have the potential to modulate intestinal microbial composition [50,51].

In the present study, the *Enterobacteriaceae* isolates derived from the cecum and cloaca of birds fed the chickpea-supplemented diet exhibited little differentiation, as only two species were identified: *Citrobacter braakii* and *Proteus mirabilis*. However, they have pathogenic potential and may pose a threat to human and animal health in certain conditions [52]. All species isolated from poultry potentially have great epidemiological importance. The active substances contained in chickpea seeds may have an impact on the abundance and differentiation of *Enterobacteriaceae* strains in the cecum and cloaca in these birds. Total phenols, polyphenols, tannins [51,53], oligosaccharides [50,51], or polyunsaturated fatty acids [54] may have a modifying effect on intestinal microbiota [55].

In favorable conditions, potentially pathogenic bacteria present in the animal gastrointestinal tract may multiply and lead to the emergence and spread of factors of resistance to some chemotherapeutics [56]. An evident sign of the presence of resistant strains in the bacterial environment is the resistance of *Enterobacteriaceae* to antimicrobial agents [57].

## 5. Conclusions

The scientific novelty of this study is that these are the first investigations in Poland to determine the impact of partial replacement of SBM protein with an alternative source of plant protein (CPR) on the intestinal histomorphology and the intestinal microbiota composition.

The inclusion of CPR in the grower and finisher diet significantly disturbed metabolism and intestinal structure. Although CPR can be a cheap source of protein, this additive does not ensure proper development of the intestinal structure, in contrast to SBM-based diets.

## Figures and Tables

**Table 1:** Components and chemical composition of starter, grower, and finisher diets for experimental male broilers [14].

Item	Diets ^1^					
	Starter (0 to 21 Days)		Grower (22 to 35 Days)		Finisher (36 to 42 Days)	
	SBM	CPR	SBM	CPR	SBM	CPR
Diet composition, %						
Maize	20.00	20.00	23.26	10.00	25.65	10.00
Wheat	42.87	42.87	44.00	35.80	44.00	40.45
Soybean meal, 46% crude protein	29.95	29.95	24.94	13.00	22.98	11.50
Chickpea, 22.5% crude protein	-	-	-	34.41	-	31.51
Soybean oil	2.59	2.59	4.56	3.45	4.68	3.73
Dicalcium phosphate	1.47	1.47	0.96	1.00	0.74	0.77
Limestone	1.30	1.30	0.86	0.80	0.63	0.59
Na_2_SO_4_	0.23	0.23	0.17	0.11	0.16	0.10
L-Lys 78%	0.39	0.39	0.24	0.32	0.20	0.29
DL-Met 99%	0.35	0.35	0.25	0.26	0.21	0.22
L-Thr 99%	0.15	0.15	0.06	0.15	0.05	0.14
NaCl	0.20	0.20	0.20	0.20	0.20	0.20
Vitamin-mineral premix	0.50 ^1^	0.50 ^1^	0.50 ^2^	0.50 ^2^	0.50 ^3^	0.50 ^3^
Values calculated						
Metabolizable energy, MJ⋅kg^−1^	12.355	12.355	13.150	13.149	13.319	13.317
Available P, %	0.470	0.470	0.350	0.350	0.299	0.300
Total Ca/Available P	2.017	2.017	2.000	1.997	2.000	2.000
Values analyzed						
Crude protein, %	21.004	21.004	18.797	18.801	17.996	17.999
Crude fiber, %	2.843	2.843	2.728	2.274	2.696	2.284
Lysine, %	1.381	1.381	1.128	1.132	1.047	1.050
Methionine, %	0.668	0.668	0.544	0.549	0.494	0.498
Methionine +cysteine, %	1.031	1.031	0.879	0.879	0.821	0.819
Threonine, %	0.925	0.925	0.756	0.756	0.716	0.716
Total Ca, %	0.948	0.948	0.700	0.699	0.598	0.600
Antinutritional factors, total in feed mixture, mg⋅g^−1^						
Tannins	6.02	6.02	5.32	3.10	5.05	2.99
Trypsin inhibitors	0.41	0.41	0.36	0.35	0.33	0.32

SBM—100% protein comes from soybean meal; CPR—the protein originated form soybean meal was replaced by 50% chickpea protein. ^1^ Added minerals and vitamins per kg of starter diet: Mn, 100 mg; I, 1 mg; Fe, 40 mg; Zn, 100 mg; Se, 0.15 mg; Cu, 10 mg; vitamin A, 15,000 IU; vitamin D3, 5000 UI; vitamin E, 75 mg; vitamin K3, 4 mg; vitamin B1, 3 mg; vitamin B2, 8 mg; vitamin B6, 5 mg; vitamin B12, 0.016 mg; biotin, 0.2 mg; folic acid, 2 mg; nicotic acid, 60 mg; pantothenic acid, 18 mg; choline, 1800 mg; coccidiostat: salinomycin; enzymes: phytase and xylanase. ^2^ Added minerals and vitamins per kg of grower diet: Mn, 100 mg; I, 1 mg; Fe, 40 mg; Zn, 100 mg; Se, 0.15 mg; Cu, 10 mg; vitamin A, 12,000 IU; vitamin D3, 5000 UI; vitamin E, 50 mg; vitamin K3, 3 mg; vitamin B1, 2 mg; vitamin B2, 6 mg; vitamin B6, 4 mg; vitamin B12, 0.016 mg; biotin, 0.2 mg; folic acid, 1.75 mg; nicotic acid, 60 mg; pantothenic acid, 18 mg; choline, 1600 mg; coccidiostat: salinomycin; enzymes: phytase and xylanase. ^3^ Added minerals and vitamins per kg of finisher diet: Mn, 100 mg; I, 1 mg; Fe, 40 mg; Zn, 100 mg; Se, 0.15 mg; Cu, 10 mg; vitamin A, 12,000 IU; vitamin D3, 5000 UI; vitamin E, 50 mg; vitamin K3, 2 mg; vitamin B1, 2 mg; vitamin B2, 5 mg; vitamin B6, 3 mg; vitamin B12, 0.011 mg; biotin, 0.05 mg; folic acid, 1.5 mg; nicotic acid, 35 mg; pantothenic acid, 18 mg; choline, 1600 mg; enzymes: phytase and xylanase.

**Table 2:** Effect of raw chickpea on histomorphometric parameters of the duodenum in 42-day-old broiler chickens ^1^.

Item	SBM	CPR	SEM	*p*-Value
Myenteron thickness, μm				
Longitudinal lamina	33.6	36.8	0.61	0.057
Transversal lamina	126.4	88.3	2.29	<0.001
Submucosa thickness, μm	18.1	15.8	0.54	0.021
Mucosa thickness, μm	1790	1539	22.19	<0.001
Villar epithelium thickness, μm	37.4	52.6	1.27	<0.001
The number of enterocytes	18.7	26.1	1.35	0.001
Villus length, μm	1492	1380	15.91	<0.001
Villus thickness, μm	117	146	2.38	<0.001
Total number of villi/mm	5.12	5.94	0.32	0.220
Crypt depth, μm	102	103	3.45	0.519
Crypt width, μm	48.3	51.3	0.91	0.073
Villus length/ Crypt depth	20.0	14.7	2,16	0.115
Intestine absorptive surface, μm^2^	26.5	21.4	0.48	<0.001
Number of crypts/mm				
Active crypts	4.92	4.23	0.24	0.154
Inactive crypts	7.89	11.0	0.73	0.025
Total crypts	12.8	15.3	0.68	0.066

^1^ Data represent the mean of 10 broiler chickens per treatment. SBM—100% protein comes from soybean meal; CPR—the protein originated form soybean meal was replaced by 50% chickpea protein; SEM—standard error of the mean.

**Table 3:** Effect of raw chickpea on histomorphometric parameters of the jejunum in 42-day-old broiler chickens ^1^.

Item	SBM	CPR	SEM	*p*-Value
Myenteron thickness, μm				
Longitudinal lamina	36.7	14.5	1.44	<0.001
Transversal lamina	235	112	7.86	<0.001
Submucosa thickness, μm	36.4	20.8	1.30	<0.001
Mucosa thickness, μm	1962	1568	23.7	<0.001
Villar epithelium thickness, μm	27.9	47.7	1.23	<0.001
The number of enterocytes	19.8	19.9	0.59	0.998
Villus length, μm	1617	1115	29.3	<0.001
Villus thickness, μm	71.8	132.6	3.65	<0.001
Total number of villi/mm	8.48	8.13	0.51	0.211
Crypt depth, μm	84.6	85.1	2.21	0.765
Crypt width, μm	41.5	44.1	1.11	0.697
Villus length/ Crypt depth	20.5	14.1	0.61	<0.001
Intestine absorptive surface, μm^2^	37.7	19.7	1.08	<0.001
Number of crypts/mm				
Active crypts	3.49	4.22	0.25	0.849
Inactive crypts	11.1	9.34	0.67	0.318
Total crypts	14.6	13.6	0.63	0.146

^1^ Data represent the mean of 10 broiler chickens per treatment. SBM—100% protein comes from soybean meal; CPR—the protein originated form soybean meal was replaced by 50% chickpea protein.; SEM—standard error of the mean.

**Table 4:** Effect of raw chickpea on histomorphometric parameters of nerve plexuses in the duodenum in 42-day-old broiler chickens ^1^.

Item	SBM	CPR	SEM	*p*-Value
Auerbach plexus				
Area, μm^2^	1836	1331	167.08	0.129
Perimeter, μm	220	190	14.55	0.368
Mean Feret diameter, μm	66.1	57.4	4.22	0.396
Min. diameter, μm	22.7	20.3	1.40	0.368
Mean diameter, μm	41. 5	35.5	1.96	0.117
Sphericity	0.09	0.09	0.01	0.554
*Meissner plexus*				
Area, μm^2^	1036	1380	128.14	0.187
Perimeter, μm	220	244	16.04	0.459
Mean Feret diameter, μm	64.9	72.2	4.68	0.452
Min. diameter, μm	11.9	13.9	0.76	0.211
Mean diameter, μm	26.3	31.5	1.59	0.102
Sphericity	0.03	0.03	0.004	0.604

^1^ Data represent the mean of 10 broiler chickens per treatment. SBM—100% protein comes from soybean meal; CPR—the protein originated form soybean meal was replaced by 50% chickpea protein; SEM—standard error of the mean.

**Table 5:** Effect of raw chickpea on histomorphometric parameters of nerve plexuses in the jejunum in 42-day-old broiler chickens ^1^.

Item	SBM	CPR	SEM	*p*-Value
Auerbach plexus				
Area, μm^2^	3289	1925	311.88	0.015
Perimeter, μm	326	212	25.97	0.035
Mean Feret diameter, μm	96.6	63.6	7.63	0.036
Min. diameter, μm	31.3	24.3	1.43	0.014
Mean diameter, μm	53.0	41.9	2.38	0.011
Sphericity	0.16	0.12	0.02	0.996
Meissner plexus				
Area, μm^2^	831	1114	72.25	0.115
Perimeter, μm	176b	246a	11.92	0.003
Mean Feret diameter, μm	52.1b	73.4a	3.58	0.003
Min. diameter, μm	13.1	11.3	0.46	0.057
Mean diameter, μm	26.8	27.1	0.86	0.842
Sphericity	0.06	0.01	0.01	<0.001

^1^ Data represent the mean of 10 broiler chickens per treatment. SBM—100% protein comes from soybean meal; CPR—the protein originated form soybean meal was replaced by 50% chickpea protein; SEM—standard error of the mean.

**Table 6:** Concentration (log cfu/g) of microorganisms in the intestinal contents of 42-day-old broiler chickens ^1^.

Item	SBM	CPR	SEM	*p*-Value
Total aerobic mesophilic bacteria count	3.36	4.29	0.432	0.039
Total count of bacteria	4.15	4.86	0.207	0.151
Total count of coliforms	2.26	3.98	0.341	0.024
Total count of fungi	1.50	1.56	0.105	0.752

^1^ Data represent the mean of 10 broiler chickens per treatment. SBM—100% protein comes from soybean meal; CPR—the protein originated form soybean meal was replaced by 50% chickpea protein; SEM—standard error of the mean.

**Table 7:** *Enterobacteriaceae* species isolated from the cecum and cloaca of broiler chickens at day 42 ^1^.

Species	SBM	CPR
Cecum		
*Citrobacter braakii*	+	++
*Citrobacter freundii*	+	-
*Escherichia coli* 1	+	-
*Escherichia coli* 2	+	-
*Enterobacter cloacae*	+	-
*Proteus mirabilis*	+	++
*Proteus vulgaris*	+	-
*Providencia rettgeri*	+	-
Cloaca		
*Citrobacter braakii*	+	++
*Citrobacter freundii*	+	-
*Escherichia coli* 1	+	-
*Escherichia coli* 2	-	-
*Enterobacter cloacae*	-	-
*Proteus mirabilis*	++	++
*Proteus vulgaris*	+	-
*Providencia rettgeri*	+	-

^1^ Data represent the mean of 10 broiler chickens per treatment. SBM—100% protein comes from soybean meal, CPR—the protein originated form soybean meal was replaced by 50% chickpea protein, - not detected, + single, ++ numerous.

**Table 8:** Sensitivity of *Enterobacteriaceae* strains isolated from the cecum and cloaca of broiler chickens at day 42 to chemotherapeutic agent percentage ^1^.

Species	SBM	CPR	*p*-Value
Cecum			
*Chloramphenicol* (30 µg/mL) ^2^			
S	70	97	
MS	15	3	0.834
R	15	0	
*Tetracycline* (30 IU) ^2^			
S	27	100	
MS	50	0	0.029
R	23	0	
*Trimethoprim/Sulfamethoxazole* (1.25 + 23.75 µg/mL) ^2^			
S	87	100	
MS	0	0	0.964
R	13	0	
*Streptomycin* (10 IU) ^2^			
S	25	100	
MS	0	0	0.011
R	75	0	
*Nitrofurantoin* (300 IU) ^2^			
S	100	93	
MS	0	7	0.936
R	0	0	
*Ampicillin* (10 µg/mL) ^2^			
S	20	100	
MS	50	0	0.013
R	30	0	
Cloaca			
*Chloramphenicol* (30 µg/mL) ^2^			
S^3^	75	100	
MS	0	0	0.598
R	25	0	
*Tetracycline* (30 IU) ^2^			
S	23	100	
MS	47	0	0.019
R	30	0	
*Trimethoprim/Sulfamethoxazole* (1.25 + 23.75 µg/mL^2^)			
S	95	97	
MS	0	0	0.659
R	5	3	
*Streptomycin* (10 IU) ^2^			
S	10	77	
MS	10	0	0.053
R	80	23	
*Nitrofurantoin* (300 IU) ^2^			
S	55	100	
MS	45	0	0.173
R	0	0	
*Ampicillin* (10 µg/mL) ^2^			
S	13	100	
MS	77	0	0.004
R	10	0	

^1^ Data represent the mean of 10 broiler chickens per treatment. ^2^ Concentration of the chemotherapeutic on the disk. SBM—100% protein comes from soybean meal, CPR—the protein originated form soybean meal was replaced by 50% chickpea protein, S—sensitive, MS—medium sensitive, R—resistant.

## Data Availability

The data that support the findings of this study are available from the corresponding author (M.K.) upon reasonable request.
